# Delayed Letters

**Published:** 1914-07

**Authors:** 


### Delayed Letters.

From many sources have come letters of bereavement and sympathy on the passing of Dr. F. E. Daniel, most of them bringing words of encouragement in new duties undertaken and responsibilities assumed. It would be a pleasure to publish all these, but space prevents it. The following letters, late in coming, express such sympathetic interest that indulgence is craved should readers of the MEDICAL JOURNAL think too much space is given to a matter so largely personal:

FROM DR. CROTHERS.

HARTFORD, CONN., July 8, 1914.

*My Dear Mrs. Daniel:*

Even at this late hour I feel impressed to write you of my appreciation of the life and work of your husband. We met in New York at the Congress on Tuberculosis, of which he was President. This gave me an opportunity to become personally acquainted, and in some respects quite intimately.

This has continued with an increasing respect and admiration. Many of his letters to me revealed his innermost thoughts, and intimations that the end was not far off. There was a subtle sort of intuitive reasoning that indicated a higher intelligence and a clearer comprehension than is given to most of us.

I chided him for his gloomy, momentary thoughts. His answer was that they were not morbid, but they were real glimpses of coming events. He was one of the very few men who was not influenced by the majority, or the popular view of events. If he thought the minority contained the real facts, he would stand by them under any circumstances. On several occasions he expressed to me a contempt for the existing views and predicted their failure, and so far he was right.

I urged him to write another book. He demurred on account of age and want of vigor. I pointed out new lines of thought, that he had developed in his last book, which could be widened and restated, and he realized this.

Dr. Daniel was a unique man and away beyond his day and generation, and the "Red Back" is the best evidence that he lived on a much higher plain than many others. I know that his words will live, not only in the memory of this generation, but in some way impress itself upon those that are coming after us. This is the highest triumph any human being can attain, by making the world better and life sweeter for those of today and tomorrow.

The "Red Back" will go on, I am sure, because it has made a place in literature, and I send my warmest condolence, and with it my most hopeful convictions that, while the Doctor has passed on, his life and memory will linger far down in the future.

Accept my warmest sympathies, and believe me,

Very sincerely yours,

T. D. CROTHERS, M. D.

FROM DR. LYDSTON.

With the passing of Dr. F. E. Daniel the profession of this country lost one of the few men in its ranks who stand for independence of thought and action and who are fearless in the cause of what they believe to be right. He never was a time-server and never subverted principle to policy. No one ever was compelled to guess where he stood. In battle, he fought in the open. His enemies respected him. In friendship he was loyal and true. His friends loved him. Be this my tribute to Daniel—high-minded gentleman, profound scholar, accomplished litterateur, and best of friends.

FROM PARKE, DAVIS & COMPANY.

DETROIT, MICH., July 6, 1914.

*Mrs. F. E. Daniel, the Texas Medical Journal, Austin, Texas.*

DEAR MRS. DANIEL: The letter from you under date of May 28th, apprising us of the death of Dr. F. E. Daniel, was in some unaccountable way filed without having reached the writer's hands for attention and reply.

We were, therefore, not aware of Dr. Daniel's death, and regret very much indeed to learn of it, and at this late date beg to express our sincere sympathy in your personal loss and also the loss which the TEXAS MEDICAL JOURNAL has sustained. If we can be of any service to you in any way, pray, have no hesitancy in commanding us.

We note that the JOURNAL will be continued under your management, and you have our best wishes for unlimited success.

With kindest regards, believe us to remain,

Very truly yours,

PARKE, DAVIS & CO.

FROM DR. ROBERTS.

TERRE HAUTE, IND., June 30, 1914.

*My Dear Mrs. Daniel:*

Seldom have I been more shocked than in the receipt of your letter written to Dr. J. A. Burnett, Hartshorne, Okla., and forwarded to me, announcing the death of your husband, Dr. Daniel, on the 14th of May, last.

I feel sure that the world has lost one of its useful men; that Texas has lost one of her brilliant citizens; that the medical profession has lost one of her great champions for all that is good and right, and I know that I have lost a personal friend.

Dr. Daniel did not have great regard for many creeds and dogmas but had a creed of his own to make a soul worth saving, knowing that an eternal just God would never allow anything to be lost that is worth saving.

Who knows that we may later meet and clasp hands with Dr. Daniel after looking forward with rapturous anticipation to the never-fading glory attainable only in the presence of the Most High.

My usual tribute in such cases is profound silence, but in this case I must depart from my usual custom and express to you my heartfelt sympathy in your bereavement.

I shall always be proud of my personal friendship for Dr. Daniel and yourself.

With very kind regards, I am,

Yours truly,

J. D. Roberts.

New York, July 6, 1914.

*Dear Mrs. Daniel:*

Pardon my delay in answering yours of June 20th. I fully intended writing to you to express sympathy on the day of my return to New York (June 1st), when I first learned that my old and highly esteemed friend had gone over to the great majority. I fear that my year in Florida has led me to absorb a trace of "manana."

It will always be one of the pleasant things of life to recall that I was permitted to know your husband for so many years.

Extending my sincere sympathy in your irreparable loss, and wishing you unprecedented success in the further management of his medico-literary pet, "The Red Back," I am,

Cordially yours,

H. M. Seem.

---

Dr. J. Hunter, who went from New York to Guadalajara, Mexico, about eight years ago, has located at Brownsville for the practice of the profession. He makes rather pessimistic reports of conditions in Mexico.

